# Ultrafast Infrared Laser Crystallization of Amorphous Si/Ge Multilayer Structures

**DOI:** 10.3390/ma16093572

**Published:** 2023-05-06

**Authors:** Alexander V. Bulgakov, Jiří Beránek, Vladimir A. Volodin, Yuzhu Cheng, Yoann Levy, Siva S. Nagisetty, Martin Zukerstein, Alexander A. Popov, Nadezhda M. Bulgakova

**Affiliations:** 1 HiLASE Centre, Institute of Physics, Czech Academy of Sciences, Za Radnicí 828, 25241 Dolní Břežany, Czech Republic; bulgakov@fzu.cz (A.V.B.);; 2 Faculty of Nuclear Sciences and Physical Engineering, Czech Technical University in Prague, Trojanova 13, 12001 Prague, Czech Republic; 3 Rzhanov Institute of Semiconductor Physics, Siberian Branch, Russian Academy of Sciences, Lavrentiev Ave, 13, Novosibirsk 630090, Russia; 4 Physics Department, Novosibirsk State University, Pirogova Street, 2, Novosibirsk 630090, Russia; 5 Coherent LaserSystems GmbH & Co. KG, Hans Boeckler Str. 12, 37079 Göttingen, Germany; 6 Institute of Physics and Technology, Yaroslavl Branch, Russian Academy of Sciences, Yaroslavl 150007, Russia

**Keywords:** silicon–germanium multilayer structures, thin films, ultrashort infrared laser annealing, selective crystallization, defect accumulation, Raman spectroscopy, laser-induced stresses

## Abstract

Silicon–germanium multilayer structures consisting of alternating Si and Ge amorphous nanolayers were annealed by ultrashort laser pulses at near-infrared (1030 nm) and mid-infrared (1500 nm) wavelengths. In this paper, we investigate the effects of the type of substrate (Si or glass), and the number of laser pulses (single-shot and multi-shot regimes) on the crystallization of the layers. Based on structural Raman spectroscopy analysis, several annealing regimes were revealed depending on laser fluence, including partial or complete crystallization of the components and formation of solid Si–Ge alloys. Conditions for selective crystallization of germanium when Si remains amorphous and there is no intermixing between the Si and Ge layers were found. Femtosecond mid-IR laser annealing appeared to be particularly favorable for such selective crystallization. Similar crystallization regimes were observed for both single-shot and multi-shot conditions, although at lower fluences and with a lower selectivity in the latter case. A theoretical analysis was carried out based on the laser energy absorption mechanisms, thermal stresses, and non-thermal effects.

## 1. Introduction

Thin solid film technologies have been proven to become a basis for the development of solar cells [1,2], solid-state electronics [3], catalysis [4], and bio-applications [5]. This is conditioned by their specific electrical, optical, and mechanical properties as well as cost efficiency, flexibility, and lightweight nature. Furthermore, the fabrication of multilayer thin film materials opens a new avenue for designing novel functional properties, which cannot be found in bulk materials [6,7]. Among multilayered materials, various combinations of silicon and germanium attract special attention since the conjunction of their optical and electrical properties makes them highly suitable in optoelectronic applications. For instance, germanium nanoinclusions in silicon are type II quantum wells or quantum dots, in which holes are localized [8]. The band gap of germanium is smaller than that of silicon (0.66 eV and 1.1 eV, respectively), and the nanoinclusions of germanium into Si-based *p-i-n* diodes can lead to improved characteristics of solar cells, primarily to expand their range to the long-wavelength region. The first such attempt was made in 2004 by Usami et al. [9] who demonstrated an extension of the quantum efficiency up to 1350 nm in structures with Ge quantum dots, although the quantum efficiency was still low. Later, based on *p-i-n* diodes with Ge nanoparticles in the *i*-layer, it was possible to extend the long-wave edge of sensitivity of photodiodes up to 1450 nm [10]. For practical use, plasma-chemical methods for the growth of such *p-i-n* diodes are promising with germanium inclusions incorporated either by plasma-chemical deposition or by pulsed laser deposition [11,12,13,14].

It is worth noting that diodes with Ge nanoparticles in the *i*-layer can be used not only in solar cells and photodetectors but also in light-emitting diodes with a spectral range of up to 1500 nm [14]. Nanostructures based on Ge–Si solid alloys can also be used in optoelectronics [8,15,16,17,18]. Thus, the development of inexpensive methods for their growth is in high demand [19]. Non-refractory materials such as Ge can be applied in “flexible electronics” devices. Recently, germanium thin films with a high carrier mobility were formed on plastic substrates using post-growth annealing at relatively low (500 °C) temperatures [20,21]. However, for substrates with a lower plasticity temperature, pulsed laser annealing for Ge crystallization looks to be more suitable, allowing additional modification of localized regions.

Pulsed laser annealing can also be efficiently used for both crystallization and nano-structuring of amorphous semiconductor films [12,13,22,23]. In the case of narrow-band semiconductor layers (e.g., Ge) alternating with a-Si layers, the laser annealing technique with infrared pulses was shown to allow selective crystallization of regions that absorb IR light [22]. In this work, we have compared single-pulse and multi-pulse irradiation regimes of ultrafast laser crystallization of Si/Ge multilayer structures (MLS) in the near-IR and mid-IR ranges and have also investigated the influence of underlying substrates on the annealing efficiency. Note that the multi-pulse regimes are particularly important for practical applications of laser annealing when fairly large, annealed areas are required and thus scanning of the laser beam over the processed area with overlapping of the irradiation spots has to be used.

This paper is organized as follows. In Section 2, a short overview of the Si/Ge MLS fabrication, experimental arrangements used for laser annealing, and the methods of post-annealing characterization are given. Section 3 starts with the presentation of the structure and optical properties of as-deposited material. Then, we report on the thresholds for modification, annealing, and ablation of the Si/Ge multilayer stacks in single- and multi-pulse irradiation regimes. Via detailed analysis of the Raman spectra, several regimes of structural changes have been determined from the formation of a germanium nanocrystalline phase, while leaving silicon layers intact to intermixing between the Si and Ge nanolayers. The effect of the substrate and the possible role of defect accumulation in laser annealing for the case of multi-shot laser action are discussed. The main findings are summarized in Section 4.

## 2. Materials and Methods

The Si/Ge MLS consisting of alternating amorphous Si and Ge nanolayers with the thickness of 40 and 15 nm, respectively, were fabricated by plasma-enhanced chemical vapor deposition (PECVD) on glass and Si (100) substrates of 1 and 0.45 mm thicknesses, respectively, as described in previous publications [13,22]. Two ultrashort laser systems were used to anneal the MLS, (1) an fs laser Astrella (Coherent, Santa Clara, CA, USA) equipped with an OPA TOPAS (Light Conversion, Vilnius, Lithuania) delivering 70 fs pulses with energy up to 400 μJ in the mid-IR range (λ = 1500 nm) and (2) a ps laser PERLA-B (HiLASE) with 1.4 ps pulses (energy up to 10 mJ) in the near-IR range (λ = 1030 nm). The annealing experiments are described elsewhere [22]. In brief, the laser pulses were focused on the MLS surface into spots with sizes 2*w*_0_ of 0.53 mm (1500 nm) and 2.1 mm (1030 nm), where *w*_0_ is the effective spot radius (1/e^2^ criterion). The peak laser fluence at the surface *F*_0_ = 2*E*_0_/π*w*_0_^2^ was varied in the range of 20–200 mJ/cm^2^. The experiments were performed under both single-shot and multi-shot irradiation conditions. In the latter case, ten individual laser pulses were applied to the same spot at a repetition rate of 1 Hz.

The structure of the as-deposited Si/Ge MLS was investigated by a transmission electron microscope (JEM-2200FS, JEOL, Akishima, Japan) using cross-sectional high-resolution transmission electron microscopy (HRTEM). Cross-sectional specimens were conventionally prepared by mechanical polishing with the use of a Leica EM TXP device (Wetzlar, Germany), followed by ion milling. The laser-produced spots on the MLS surface were analyzed by an optical microscope (Olympus BX43, Shinjuku, Japan) and scanning electron microscopy (SEM TESCAN MIRA3 LMH, Brno, Czech Republic).

Raman spectra were recorded in the back-scattering geometry using a 514.5 nm fiber laser line as an excitation source. The T64000 spectrometer with a spectral resolution of 2 cm^−1^ (Horiba Jobin Yvon, Lille, France) was used. The analysis of scattered light polarization was not carried out. The setup for microscopic studies of Raman scattering (micro-Raman) was utilized. To avoid heating of the MLS under the excitation laser beam during the Raman measurement, the sample was placed below the focus and the spot size was 10 μm, i.e., much smaller than the annealed spot size. Therefore, the very central spot part, irradiated at peak fluence *F*_0_, was analyzed in the Raman measurements. The light transmission of the sample in the visible and near-IR ranges was studied using a Shimadzu-3600 spectrophotometer (Kyoto, Japan).

## 3. Results and Discussion

**Figure 1:**
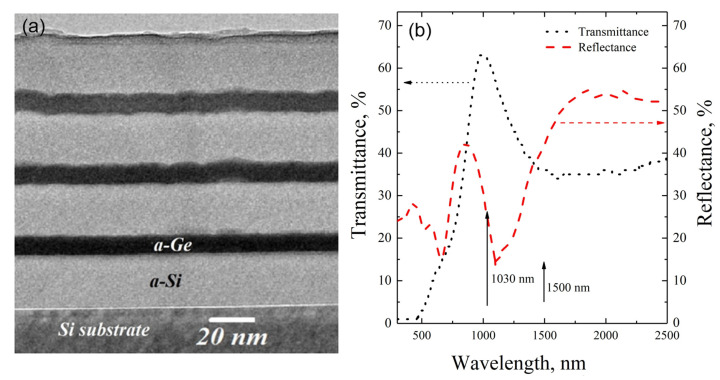
The cross-sectional TEM image (**a**) and transmission and reflectance spectra (**b**) of as-deposited Si/Ge MLS.

Figure 1a shows a cross-sectional TEM image of the as-deposited Si/Ge MLS. The dark stripes correspond to the 15 nm thick Ge layers, and the lighter areas are the Si layers. Figure 1b shows the light transmission and reflectance spectra of the MLS. It is seen that the structure is semitransparent for both used wavelengths (shown by the arrows). The transmission maximum at about 1000 nm is due to interference in the MLS.

**Figure 2:**
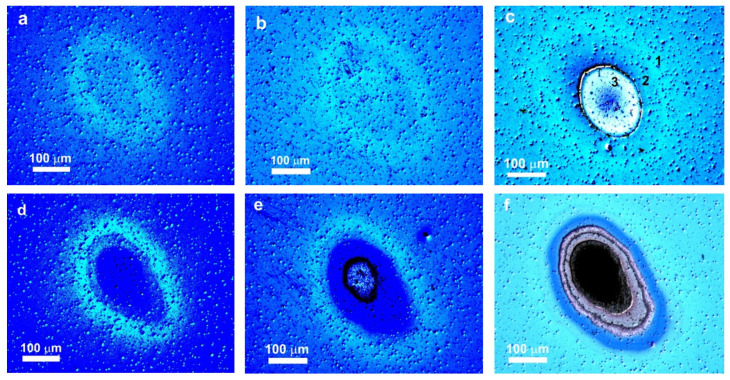
Nomarski optical images of spots produced on the MLS surface by single (**a**–**c**) and ten (**d**–**f**) mid-IR pulses at fluences of 77 (**a**,**d**), 95 (**b**,**e**), and 145 mJ/cm^2^ (**c**,**f**). The spot regions related to (1) modification, (2) damage/annealing, and (3) ablation are shown in (**c**) by the corresponding numbers.

Figure 2 shows typical images of spots produced on the MLS surface (Si substrate) by mid-IR laser pulses in different fluence regimes under single- and 10-shot conditions. The spots have a characteristic three-zone structure often observed at the surface of bulk crystalline silicon laser-irradiated by ultrashort Gaussian pulses [24,25,26]. The three zones can be readily distinguished, as shown in Figure 2c, and include, according to [22,25], an external modification region 1 (related to surface oxidation), a middle damage/annealing region 2, and a central region 3 produced due to ablation. At low laser fluences, below the ablation threshold, region 3 is not observed (Figure 2a,b,d). When comparing single-shot and ten-shot irradiation regimes (top and bottom layers in Figure 2, respectively), one can see that the laser-induced damage and ablation are considerably stronger in the latter case due to incubation effects [25,27]. Thus, 10 shots at 95 mJ/cm^2^ result in ablation of the top Si layer, whereas no ablation with a single shot at this fluence is observed (compare Figure 2b,e). At higher fluence, when only the top layer is ablated by a single laser shot, several layers are removed under multi-shot conditions (Figure 2c,d). Similar images were observed for the MLS on glass substrates under identical irradiation conditions.

**Figure 3:**
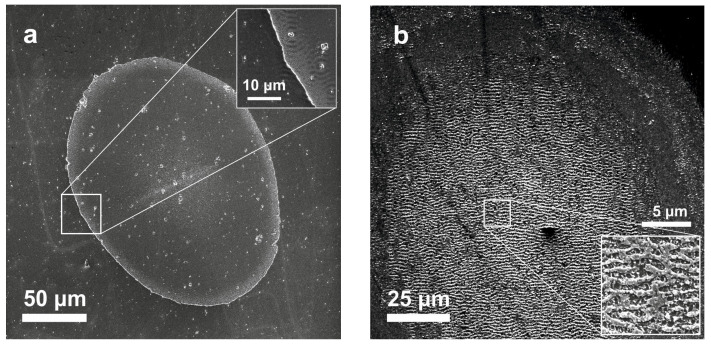
SEM images of spots produced on the MLS surface by a single mid-IR shot at 145 mJ/cm^2^ (**a**) and ten shots at 120 mJ/cm^2^ (**b**). The insets show at higher magnifications the boundary between the ablation and damage regions in (**a**) and the structured central spot part in (**b**).

Examples of SEM images of spots produced on the MLS surface on a Si substrate by single and ten mid-IR laser shots in the ablative regime are shown in Figure 3. Under single-shot conditions, the central ablation region is fairly smooth and has a well-defined, sharp boundary (Figure 3a). However, nucleation of laser-induced periodic surface structures (LIPSS) is seen around the surface defects in the form of semi-concentric patterns (inset in Figure 3a) [28]. In the multi-shot regime (Figure 3b), the central ablation region is fine structured with LIPSS characteristic features [29].

**Figure 4:**
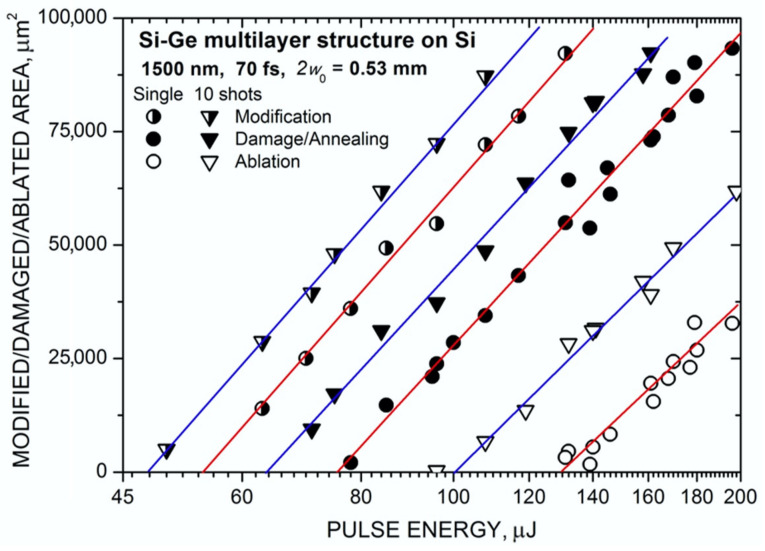
Areas of different zones within the spots produced by single and ten mid-IR laser pulses on the MLS surface as a function of pulse energy. The lines correspond to the least-square fits using Equation (1).

The obtained optical images of the spots are analyzed using the standard procedure for Gaussian beams when the area *S* of a specific zone within the spot is related to the pulse energy *E*_0_ by the formula [30,31]:

(1) $${S=\frac{\pi \omega_{0}^{2}}{2}\ln(E}_{0}/E_{th})$$

where *E*_th_ is the corresponding threshold energy (modification, damage, ablation). By measuring the *S* value versus the pulse energy and extrapolating to zero, one can determine the threshold fluence *F*_th_ = 2*E*_th_/π*w*_0_^2^ for every spot region. Figure 4 shows such dependencies for different zones produced by mid-IR laser pulses at the MLS surface on a Si substrate for both single- and 10-shot regimes. Nearly identical results were obtained for the Si/Ge MLS on a glass substrate. All the data are well approximated by Equation (1) with an identical slope in the semi-logarithmic plot corresponding to the effective spot diameter 2*w*_0_ = 0.53 mm. Similar data obtained with near-IR laser pulses resulted in a larger spot diameter 2*w*_0_ = 2.1 mm and almost the same threshold values for both single and ten shots. The thresholds measured for all the studied irradiation conditions are summarized in Table 1.

**Figure 5:**
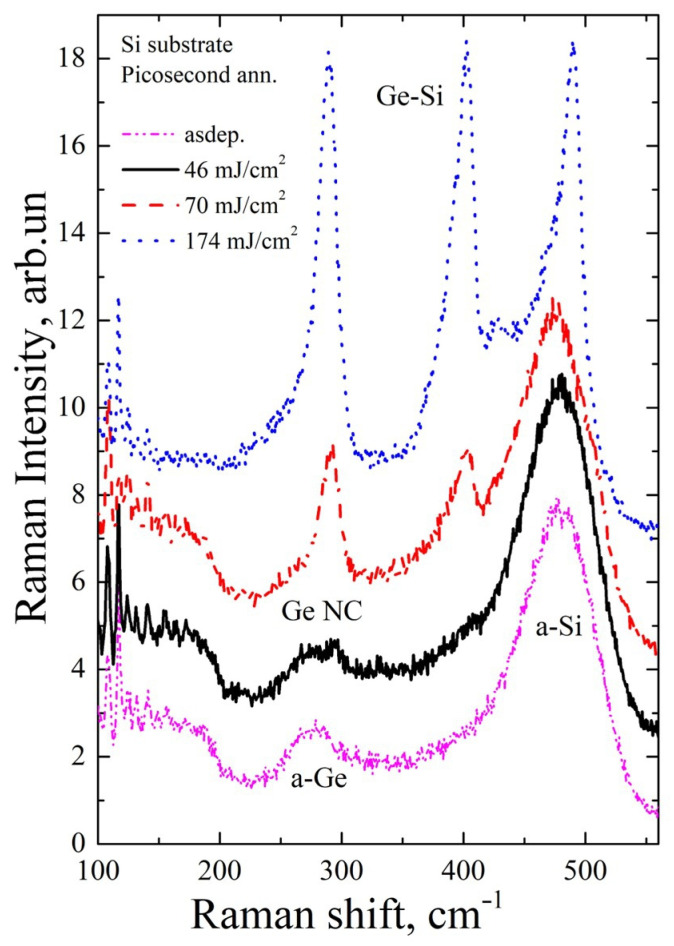
Raman spectra of the MLS at a Si substrate irradiated by single near-IR pulses.

**Figure 6:**
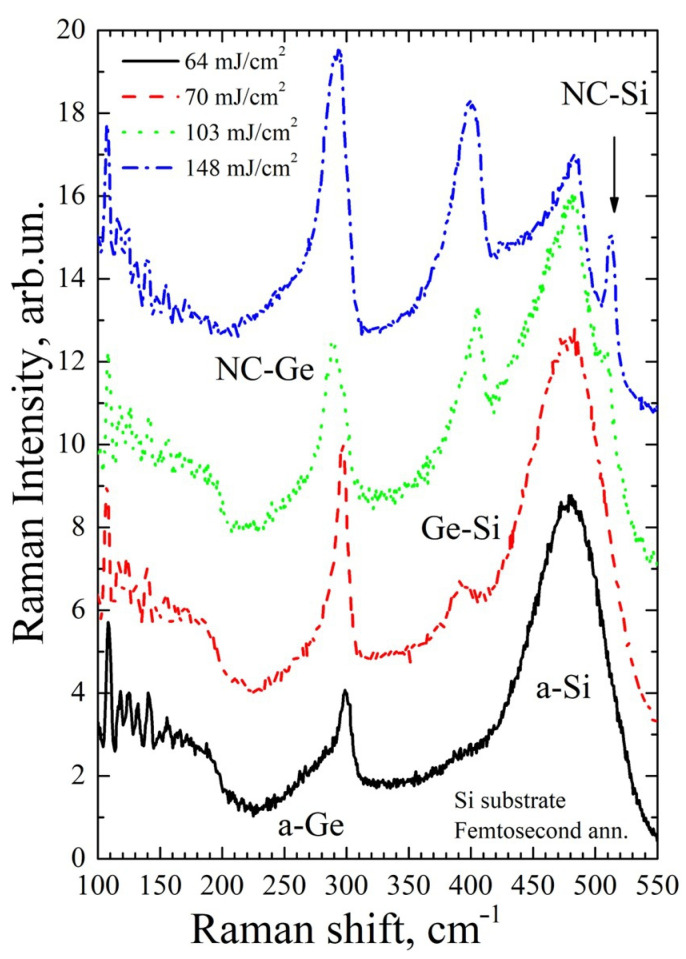
Raman spectra of the MLS at a Si substrate irradiated by single mid-IR pulses.

**Figure 7:**
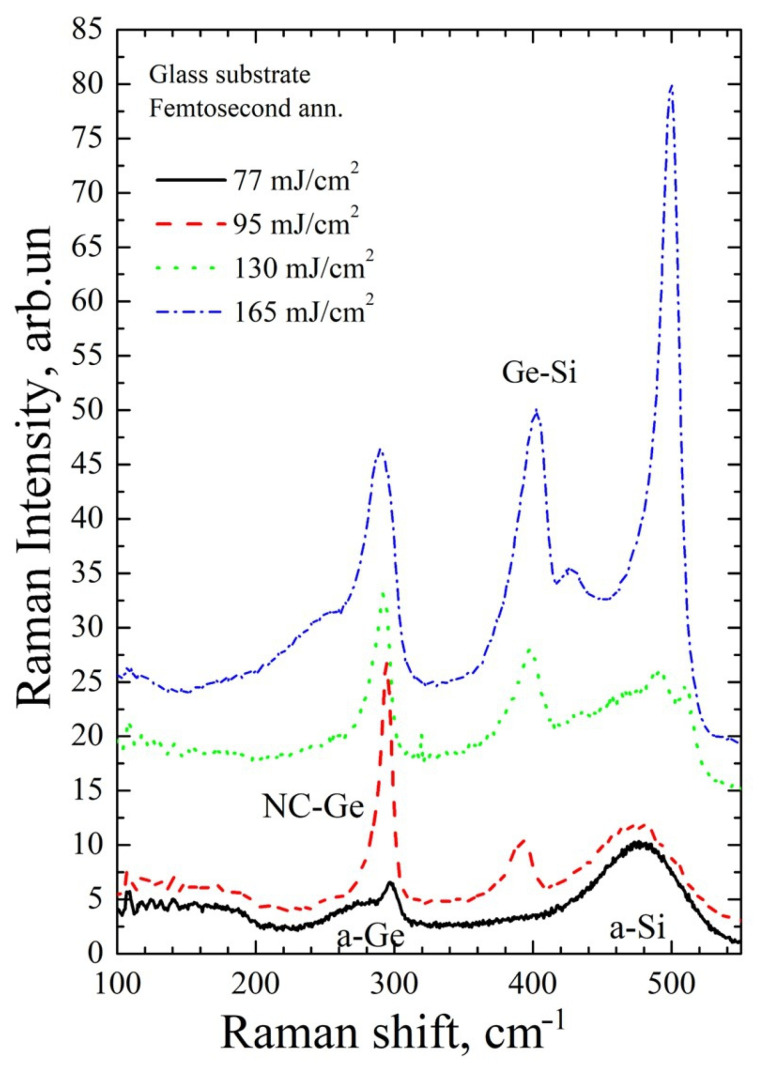
Same as Figure 5 but for a glass substrate.

**Figure 8:**
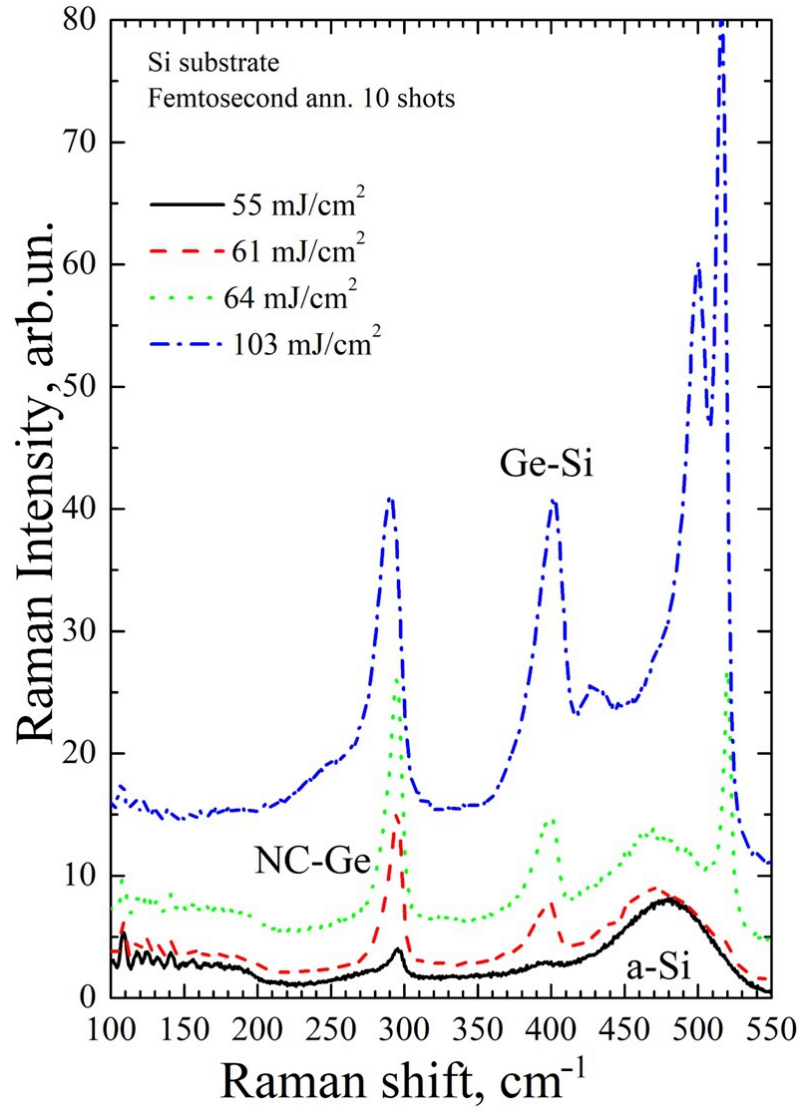
Same as Figure 5 but for 10 shots.

Figure 5, Figure 6, Figure 7 and Figure 8 show Raman spectra of the Si/Ge MLS on glass and Si substrates, irradiated by near-IR and mid-IR laser pulses at different fluences (one or ten shots). The used laser fluence range covers conditions from the early beginning of Ge crystallization up to intermixing of the Si/Ge layers and Si crystallization.

Figure 5 shows Raman spectra of both as-deposited and annealed samples on a Si substrate. In the Raman spectrum of the non-irradiated MLS (magenta dot-dot-dash line in Figure 5), there are broad bands at ~275 cm^–1^ and ~480 cm^–1^. These bands are related to the maximum density of vibrational states of amorphous Ge [32] and Si [33], respectively. It is known that the frequencies of Ge–Si bond vibrations are ~400 cm^–1^ [34,35,36]. Note that the experimental spectrum of the as-deposited sample does not contain a band corresponding to Raman scattering by vibrations of these bonds. This indicates that the concentration of the Ge–Si bonds is too small to be detected, although they certainly should be present at the Ge/Si interfaces. When ps near-IR annealing with fluences less than 40 mJ/cm^2^ (i.e., below the modification threshold, see Table 1) was applied, no modification to the structure was observed in the Raman spectrum; both layers remained amorphous.

After ps annealing with near-IR pulses at *F*_0_ = 46 mJ/cm^2^ (black, solid line in Figure 5), the beginning of crystallization of Ge layers is observed. This manifests itself as the appearance of a relatively narrow peak with a position of about 293 cm^−1^. It is known that the frequency of the long-wave optical phonons in bulk crystalline Ge is about 301 cm^−1^ [37]. According to the phonon confinement model (PCM) [38], the Raman scattering peak of Ge nanocrystals (NCs) shifts towards lower frequencies with decreasing nanocrystal size. According to the PCM, the observed shift of 8 cm^−1^ (fluence 46 mJ/cm^2^) corresponds to the nanocrystal diameter of 2 nm which is far less than the thickness of the germanium layers. There is still a significant “amorphous” peak in the spectrum, which indicates that most of the Ge layers remained amorphous.

As the laser fluence increases, the fraction of the crystalline phase increases (70 mJ/cm^2^, red dashed line in Figure 5). However, at the same time, a peak arises from scattering on Ge–Si bond vibrations (~400 cm^–1^). This indicates that a Ge–Si solid alloy is formed at the Ge–Si interfaces but the layers of silicon that are not involved in mixing with germanium remain amorphous. In the case of the formation of Ge–Si solid alloys, it is impossible to determine the size of Ge or Ge–Si nanoinclusions. With further increasing laser fluence, almost complete intermixing between the Ge and Si layers occurs (174 mJ/cm^2^, blue dotted line in Figure 5). By comparing the relative intensities of the Ge–Ge and Ge–Si peaks, we can estimate the fractions of Ge and Si in the Ge_x_Si_(1−x)_ NCs using the following formulas [34]:

(2) $$\frac{I_{SiSi}}{I_{GeSi}}=A\frac{\left(1-x\right)}{2x}$$

(3) $$\frac{I_{GeGe}}{I_{GeSi}}=B\frac{x}{2\left(1-x\right)}$$

Here *I*_GeGe_, *I*_GeSi_, and *I*_SiSi_ are the integral intensities of the Raman signals from the corresponding bond oscillations. It is known [34] that the coefficients *A* (ratio of Raman cross-sections for Si–Si and Ge–Si bonds) and *B* (ratio of Raman cross-sections for Ge–Ge and Ge–Si bonds) depend on the stoichiometry parameter *x* in Ge_x_Si_(1−x)_ solid alloys. It should be noted that if the Ge and Si layers are mixed homogeneously, then, by the ratio of the layer thicknesses, the parameter of the stoichiometric composition *x* of the solid alloy would be 0.27. According to Equation (2), *x* is close to 0.5 while, according to Equation (3), *x* ≈ 0.4. This indicates that silicon layers are not completely mixed with germanium, and the unmixed silicon remains mostly amorphous.

An analysis of the Raman spectra of the Si/Ge structures on the Si substrate after annealing with fs mid-IR pulses (Figure 6) points out that the fluence dependences of the structural transformation are quite similar to those obtained at ps irradiation. However, at this irradiation condition, a regime has been found in which a significant part of the Ge layers is crystallized without detectable intermixing of the Ge and Si layers and crystallization of the Si layers (fluence 64 mJ/cm^2^, the black solid line in Figure 6). In this case, the position of the peak from germanium nanocrystals is 299 cm^−1^, which corresponds to their sizes of about 4.5–5 nm. When the fluence increases by only about 10%, the Ge layers begin to mix with silicon (fluence 70 mJ/cm^2^, red dashed line in Figure 6). With further increasing fluence (103 mJ/cm^2^, green dotted line in Figure 6), intermixing becomes more pronounced while almost all silicon remains amorphous. One can notice only a weak peak at 510 cm^−1^, which corresponds to nanocrystalline silicon (NC-Si). The PCM is also applicable for silicon nanocrystals and, according to estimates, the size of silicon NCs in this case is 2–2.2 nm [39]. At even higher fluences, and hence stronger intermixing, a layer of Ge–Si solid alloy with inclusions of nanocrystalline silicon is formed (148 mJ/cm^2^, blue dot-dashed line in Figure 6). However, in this case, in addition to the peaks from the Ge–Si solid alloy, there is a more pronounced peak from silicon nanocrystals with a position of 512 cm^−1^. The size of silicon nanocrystals in this case is about 2.5 nm [39].

Figure 7 shows Raman spectra of Si/Ge MLS on a glass substrate after annealing with fs mid-IR pulses. No fundamental differences between the silicon and glass substrates are found concerning the transformation of the structure for this wavelength. Several regimes with increasing laser fluence are also observed for the glass substrates. At low fluences, although higher ones compared to the Si substrates, the Ge layers are partially crystallized while the Si layers remain amorphous without noticeable intermixing of Ge and Si (77 mJ/cm^2^, black solid line in Figure 7). The Ge layers are almost fully crystallized without crystallization of the Si layers but some Ge–Si intermixing occurs (fluence 95 mJ/cm^2^, red dashed line in Figure 7). Complete crystallization of the Ge layers is achieved but with partial crystallization of the Si layers and Ge–Si intermixing (130 mJ/cm^2^, green dot line in Figure 7). Almost complete intermixing of the Ge and Si layers is observed with further increase in fluence (165 mJ/cm^2^, blue dot-dashed line in Figure 7). It should be noted that in the last case, the Ge and Si layers are mixed almost homogeneously because the stoichiometry parameter *x* of the Ge_x_Si_(1−x)_ solid alloy is about 0.32, according to Equation (3).

However, some noticeable influence of the substrate type has been observed. Laser fluences required to achieve identical crystallization regimes are different for the multilayer structures on silicon and glass. This can be due to the influence of several factors. The main factor is seen in the substrate reflectivity. As the generated free electron density in Ge layers is sub- or near-critical [22], the laser light penetrates toward the MLS–substrate interface. Applying the multilayer reflection formalism [40], we found that, for the 1500 nm wavelength, only ~4% of laser light reflects from the MLS–glass substrate system. As for the Si substrate, almost 40% of laser light penetrating through the MLS reflects and propagates through already excited Ge layers where additional absorption takes place via photoionization, possibly due to the collisional multiplication of electrons. Although these reflectivity estimates were performed for crystalline layers, the trend should be the same for the amorphous samples. Other factors that can contribute to the observed substrate effect are the thermal conductivity of glass, which is lower than that of silicon, and different thermal expansion coefficients (8.5 × 10^−6^ K^−1^ for glass and 2.6 × 10^−6^ K^−1^ for silicon at 20 °C [41]) that may be important if considering the elastic energy effects in phase transitions.

Figure 8 shows Raman spectra of Si/Ge MLS on a Si substrate after mid-IR annealing with ten laser shots. It is seen that all the above-determined crystallization regimes can also be achieved; however, they are achieved at lower laser fluences in agreement with the observed lower damage and ablation threshold values for multi-shot irradiation conditions (Table 1). Comparing the data in Figure 6 and Figure 8, one can note that with 10 pulses it was possible to crystallize germanium already at 55 mJ/cm^2^ (black solid line in Figure 7), which is ~14% lower than the minimal fluence needed to observe Ge crystallization after a single 1500 nm pulse (64 mJ/cm^2^, Figure 5). This decrease in the Ge crystallization thresholds is in excellent agreement with the difference in the damage threshold values for single and ten laser shots (Table 1). According to the PCM, for the peak position at 295 cm^−1^, the nanocrystal diameter can be estimated to be ~2.7 nm. However, one may notice a slight sign of Si–Ge bonds in this irradiation regime which were not observed near the single-shot crystallization onset. When the fluence increases by 11%, intermixing of the Ge layers with silicon becomes pronounced (61 mJ/cm^2^, red dashed line in Figure 8). With a further slight increase in fluence (only by ~5%), crystallization of the silicon layers is initiated (64 mJ/cm^2^, green dot line in Figure 8). At 103 mJ/cm^2^, partial mixing of the Ge and Si layers is observed, with crystallization of the remaining unmixed silicon (blue dot-dashed line in Figure 8).

The time between pulses of 1 s is long enough for the entire structure to cool down completely [42,43]. Thus, the reduction in the Ge crystallization threshold from 64 J/cm^2^ at single-pulse irradiation to 55 J/cm^2^ at 10 laser pulses (Figure 6 and Figure 8) cannot be explained by the heat accumulation effect. To understand the difference between single- and multi-pulse laser annealing of the Si/Ge MLS, fundamental aspects of laser energy coupling should be applied [22]. It was found that the main mechanism governing the crystallization of the Ge layers in the considered irradiation regimes is single-photon absorption with a noticeable contribution of two-photon ionization. It leads to the generation of free electrons, which can absorb additional energy from the laser beam. Thermalization of free electrons with the lattice and their recombination proceed in the timescale of several picoseconds. Depending on laser fluence, different scenarios can be realized, namely, (a) no visible modification of the MLS, (b) partial melting of the Ge layers, (c) their complete melting with heat transfer to the Si layers, and (d) melting of silicon at the Ge–Si interfaces where intermixing between layers’ species occurs. As mentioned above, between successive pulses the heat transfer cools down the laser-affected MLS zone to practically the initial temperature. According to the analytical theory provided in [22], the levels of the initial heating of germanium irradiated with 1500 nm femtosecond laser pulses and the associated stresses decrease nonlinearly with reducing laser fluence due to a noticeable contribution of two-photon ionization (e.g., from ~795 K and ~0.71 GPa at 64 J/cm^2^ to ~710 K and ~0.58 GPa at 55 J/cm^2^, as can be estimated using the approach [22]). However, it is known that sequential pulses can lead to the accumulation of defect states, which are active in photon absorption [27]. Additionally, the laser-induced stresses are still substantial even at fluences below the melting threshold value, which can lead to some distortion of the metastable amorphous matrix towards a more equilibrium crystalline state. Such minor modifications, being too small after a single pulse to be detectable by Raman spectroscopy, can be accumulated from pulse to pulse, yielding, after a certain number of pulses, the appearance of detectable crystallites. Although the accumulation effects in the amorphous semiconductors are still insufficiently studied, such a scenario of reducing the overall threshold after multiple laser pulse actions seems to be most likely to occur.

## 4. Conclusions

Laser crystallization of amorphous Si (40 nm)/Ge (15 nm) multilayer structures (MLS) on Si and glass substrates by laser annealing with femtosecond mid-IR and picosecond near-IR pulses was investigated. Several regimes of crystallization were found for both near-IR and mid-IR pulses with increasing laser fluence involving partial or complete crystallization of only germanium or both components with a different degree of intermixing between the Si and Ge layers. In particular, conditions for selective crystallization of germanium without detectable Si–Ge intermixing were revealed. The regimes with one- and ten-laser shots were compared and the mechanisms of the ultrafast infrared laser annealing have been discussed. Finally, we underlined that femtosecond laser annealing at wavelengths toward the mid-IR spectral range is more favorable for the selective crystallization of germanium in Ge/Si MLS when compared to picosecond pulses. We anticipate that, at longer pulses, the bremsstrahlung light absorption leads to higher electron energies in the Ge layers. Such electrons generated near Ge–Si interfaces may affect interface-adjacent Si atoms, resulting in their destabilization, bonding to Ge atoms, and Ge–Si intermixing. Additionally, single pulse annealing yields, as a whole, a better quality of selective Ge crystallization. We believe that the proposed selective crystallization technique can be efficiently used for the production of nanocrystals of narrow-gap semiconductors in an amorphous silicon matrix that will improve parameters of silicon-based devices such as solar cells, photodetectors, and light-emitting diodes [2,3,12,14]. This work is currently in progress.

## Figures and Tables

**Table 1:** Thresholds for modification (*F*_th,m_), damage (*F*_th,d_), and ablation (*F*_th,a_) of the Si/Ge MLS by single and ten ultrashort laser pulses.

Irradiation Conditions	*F*_th,m_, mJ/cm^2^		*F*_th,d_, mJ/cm^2^		*F*_th,a_, mJ/cm^2^	
	1 Shot	10 Shots	1 Shot	10 Shots	1 Shot	10 Shots
1500 nm, 70 fs	50	45	70	60	115	90
1030 nm, 1.4 ps	45	40	65	55	110	85

## Data Availability

Data are contained within the article.
